# Supplementary material for: Identifying an optimal dihydroartemisinin-piperaquine dosing regimen for malaria prevention in young Ugandan children
Source: Nat Commun. 2021 Nov 18;12:6714. doi: 10.1038/s41467-021-27051-8 (PMC8602248; doi:10.1038/s41467-021-27051-8)
Supplement: Supplementary file 1 — Supplementary Information [file 41467_2021_27051_MOESM1_ESM.pdf]

**Identifying an optimal dihydroartemisinin-piperaquine dosing regimen for malaria prevention in young Ugandan children**

## Supplemental Tables

**Supplementary Table 1.** Simulated DP dosing regimens

| **DP Regimen** | **Dosing bands** |
|---|---|
| Protocol regimen based on manufacturer package insert | <6 kg: DHA/PPQ 10/80 mg daily x 3 days<br>6-<11 kg: DHA/PPQ 20/160 mg daily x 3 days<br>11-<15 kg: DHA/PPQ 30/240 mg daily x 3 days<br>15-<20 kg: DHA/PPQ 40/320 mg daily x 3 days |
| WHO 2015 regimen | <8 kg: DHA/PPQ 20/160 mg daily x 3 days<br>8-<11 kg: DHA/PPQ 30/240 mg daily x 3 days<br>11-<17 kg: DHA/PPQ 40/320 mg daily x 3 days<br>17-<25 kg: DHA/PPQ 50/480 mg daily x 3 days |
| Age-based 6 & 18 months | 2-<6 months of age: DHA/PPQ 20/160 mg daily x 3 days<br>6-<18 months of age: DHA/PPQ 30/240 mg daily x 3 days<br>18-24 months of age: DHA/PPQ 40/320 mg daily x 3 days |

**Supplementary Table 2. PK-QTc model parameters**

| Parameter | Value (% RSE) | Interindividual Variability (% RSE) |
|---|---|---|
| N (subjects) | 32 | |
| Pre-drug QTcB (msec) | 410 (0.5) | 1.4% (41) |
| $\Theta_{Slope}$/1000 (msec/ng/mL) | .0463 (45) | - |

## Supplemental Figures

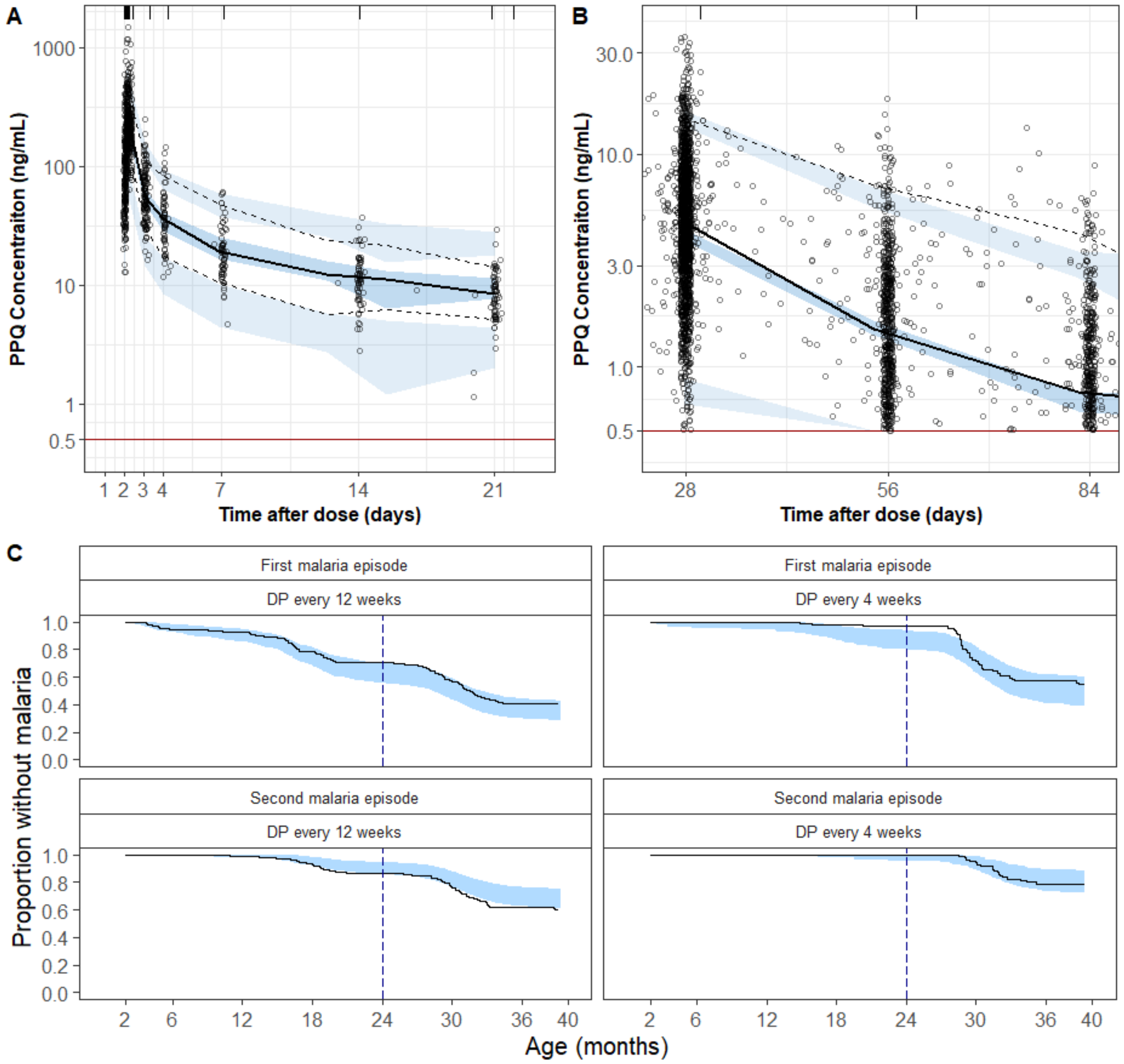


**Supplementary Figure 1.** Visual predictive check (VPC) of final pharmacokinetic/pharmacodynamic (PK/PD) model. (A) VPC of intensive piperaquine (PPQ) data through 21 days after dose, with sampling beginning after the last daily dose (day 2) and (B) VPC of sparse PPQ data collected 28, 56, and 84 days after DP. Points are observed PPQ concentrations, solid line indicates median of observed data, and dashed lines indicate the 5%

and 95% confidence intervals of observed data. Shaded areas indicate 5%, 50% and 95% of simulated data from the final PPQ PK model. Red line at 0.5 ng/mL indicates the lower limit of quantification for the PPQ assay.  (C) VPC for PK/PD repeated time to event model for incident malaria. Solid line indicates time from start of the study to labeled malaria episode, shaded areas describes 95% of the simulated data.

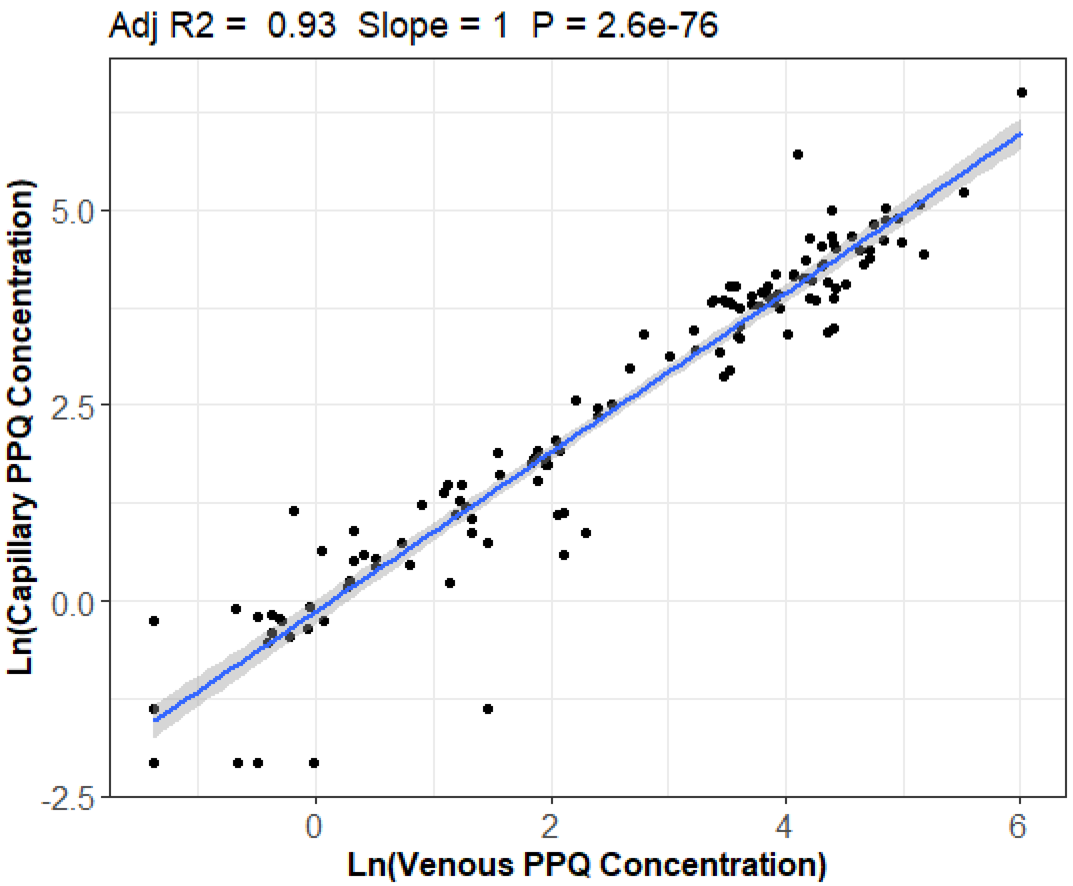


**Supplementary Figure 2**. Venous and capillary PPQ concentration correlation. The slope and statistical significance were determined by linear regression. The regression line is in blue, with the 95% confidence interval of the estimate shaded in grey. Paired samples were available from 70 children who participated in the study.

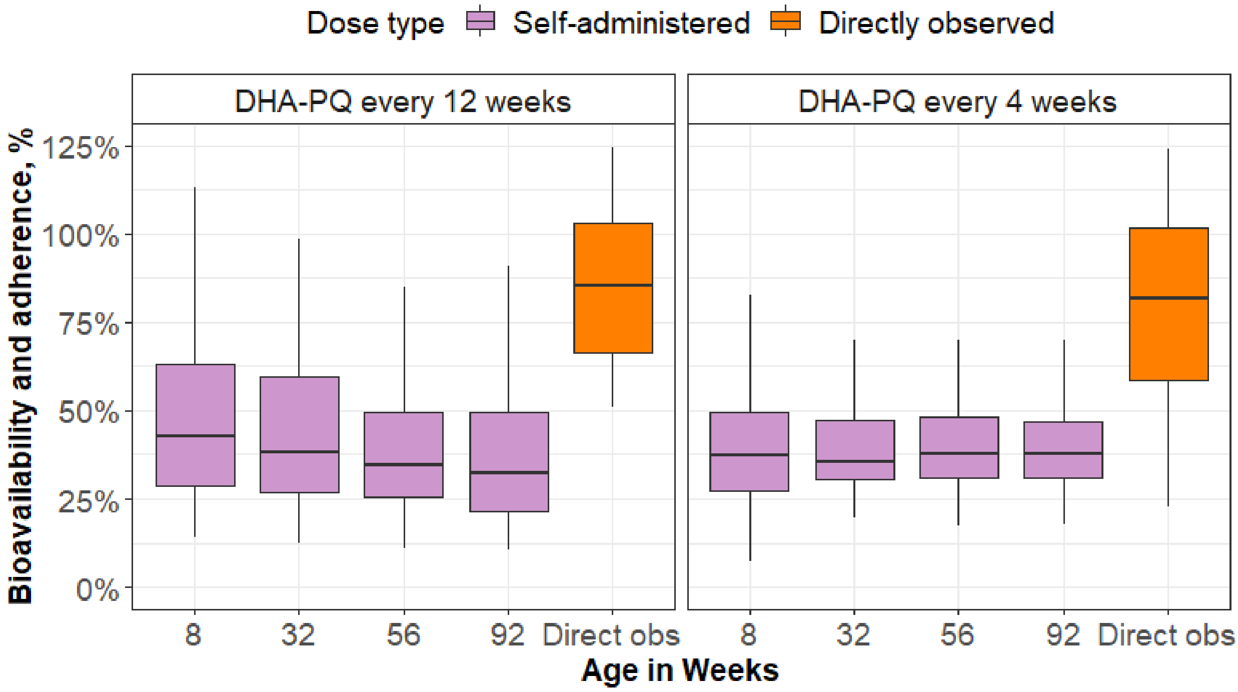


**Supplementary Figure 3.** Comparison of estimated bioavailability of piperaquine (PPQ) between directly observed and non-directly observed (self-administered) treatment courses. When the first of three dihydroartemisinin-piperaquine (DP) doses was directly observed (self-administered), the boxplots are shaded in purple and when all three doses were directly observed the boxplot is shaded orange. Boxes indicate PPQ relative bioavailability for 25% (minima), 50% (center) and 75% (maxima) of the population, and vertical bars represent relative bioavailability levels for 95% of the population.  Data are from 184 children in the DP every 12 week arm and 96 children in the DP ever 4 week arm.

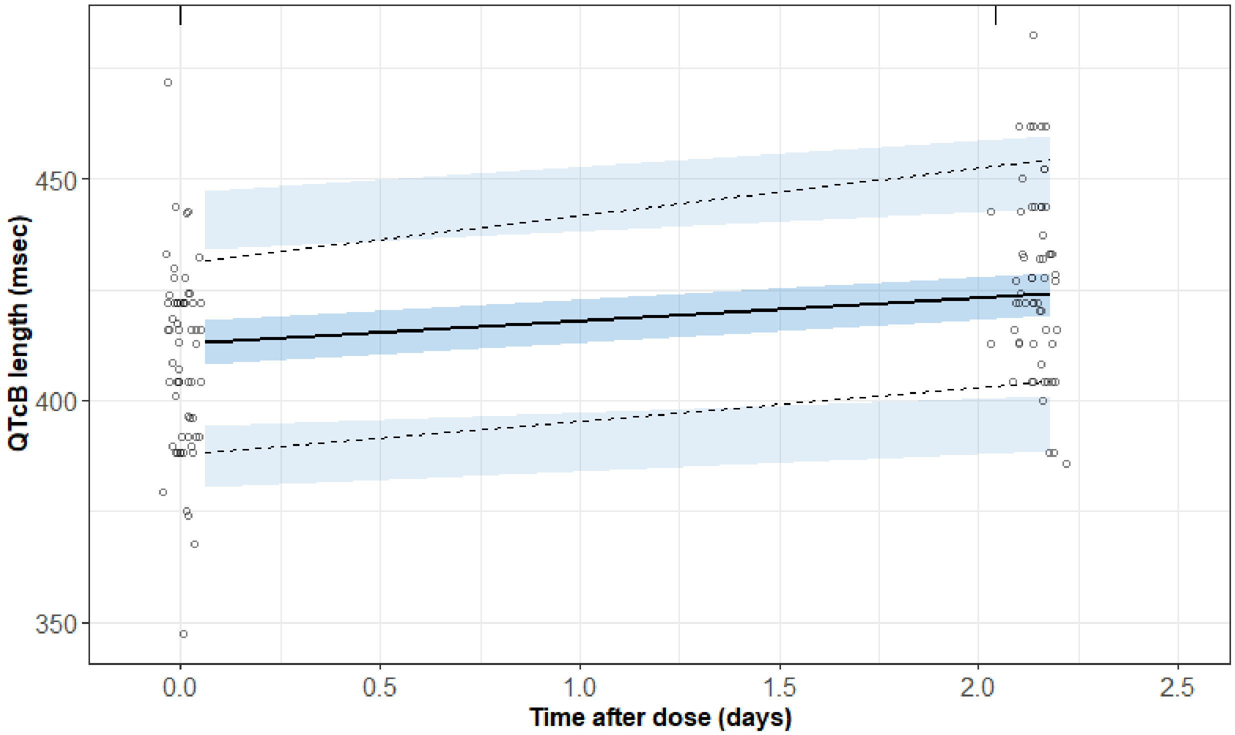


**Supplementary Figure 4.** Visual predictive check for the pharmacokinetic-corrected QT interval by Bazett's formula (PK-QTcB) model. Dots indicate observed data, and lines indicate median, 10% and 90% of the population. Shaded areas indicate the 10%, 50%, and 90% ranges of the simulated data. Data are from the 32 participants from the intensive pharmacokinetic substudy.

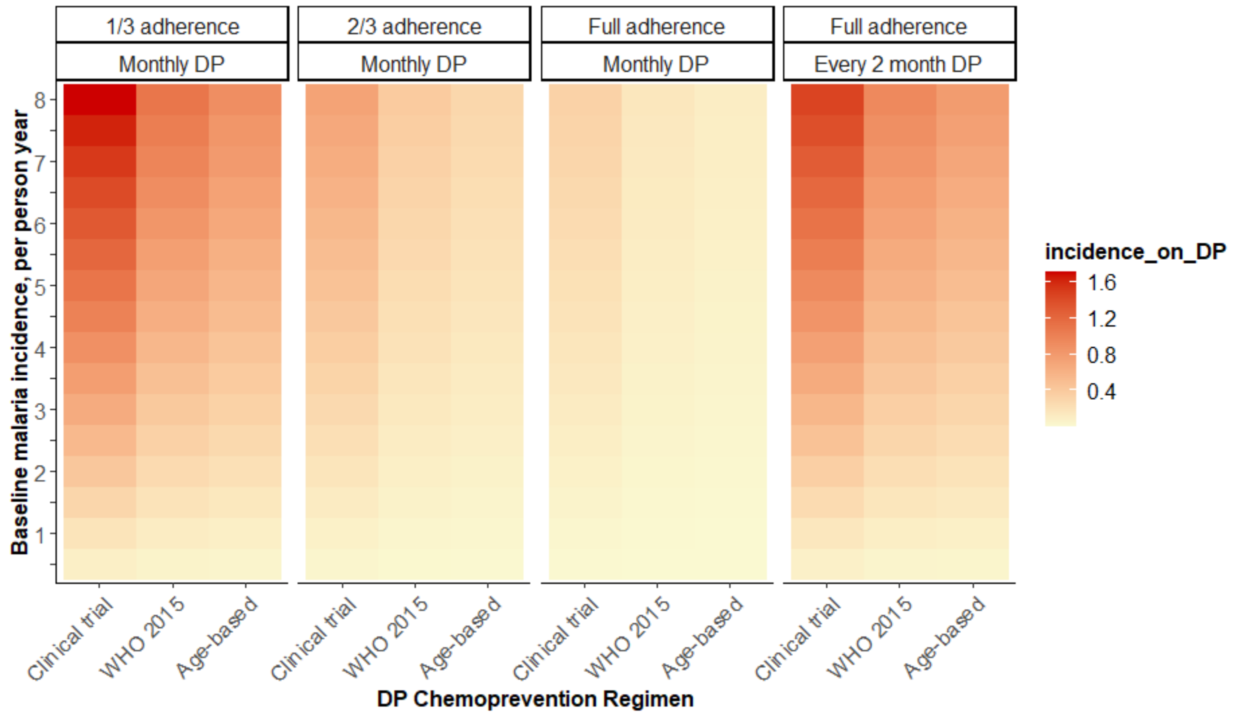


**Supplementary Figure 5.** Predicted impact of adherence and dihydroartemisinin-piperaquine (DP) regimen on malaria incidence with different chemoprevention regimens, including by dose and frequency. 1/3 adherence indicates bioavailability observed for non-directly observed therapy in the study, 2/3 adherence indicates a bioavailability midpoint between the directly and non-directly observed population, and full adherence indicates the bioavailability observed in the directly observed therapy group. Simulations were conducted using the 10,000 simulations of the 280 children from the study population and incidence calculated between 2 and 24 months of age. Age-based dosing indicates daily piperaquine doses as follows (<6 months = 160 mg; 6-<18 months = 240 mg; 18-26 months = 320 mg).

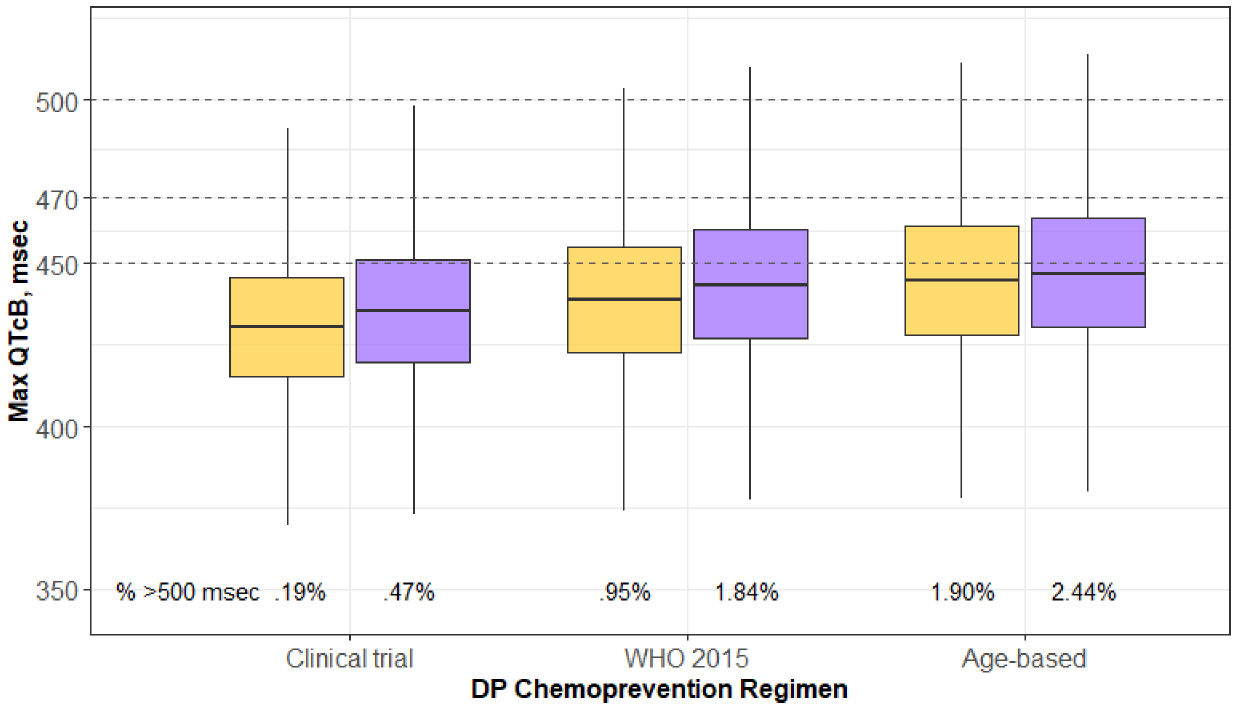


**Supplementary Figure 6.** Predicted maximum corrected QT interval using Bazett's formula (QTcB prolongation) by dihydroartemisinin-piperaquine (DP) regimen. Maximum piperaquine (PPQ) concentrations predicted with the currently approved WHO 2015 regimens and the proposed age-based dosing regimens were higher than the maximum PPQ concentrations used to develop the PK-QTc model. The gold boxes indicate a malnourished population at the start of chemoprevention with WAZ≤-2 and purple indicates a better nourished population with WAZ>-2 at the start of chemoprevention. The boxes indicate the QTcB for 25% (minima), 50% (center) and 75% (maxima) of the population, and vertical bars represent the QTcB for 95% of the population. Data are from 10,000 simulations of demographic information from 280 children.
