# Supplementary material for: Identifying an optimal dihydroartemisinin-piperaquine dosing regimen for malaria prevention in young Ugandan children
Source: Nat Commun. 2021 Nov 18;12:6714. doi: 10.1038/s41467-021-27051-8 (PMC8602248; doi:10.1038/s41467-021-27051-8)
Supplement: Supplementary file 3 — Reporting Summary [file 41467_2021_27051_MOESM3_ESM.pdf]

nature portfolio

Corresponding author(s): Rada Savic

Last updated by author(s): Oct 11, 2021

Reporting Summary

Nature Portfolio wishes to improve the reproducibility of the work that we publish. This form provides structure for consistency and transparency in reporting. For further information on Nature Portfolio policies, see our Editorial Policies and the Editorial Policy Checklist.

Statistics

For all statistical analyses, confirm that the following items are present in the figure legend, table legend, main text, or Methods section.

n/a	Confirmed	
☐	☒	The exact sample size (*n*) for each experimental group/condition, given as a discrete number and unit of measurement
☐	☒	A statement on whether measurements were taken from distinct samples or whether the same sample was measured repeatedly
☐	☒	The statistical test(s) used AND whether they are one- or two-sided<br>*Only common tests should be described solely by name; describe more complex techniques in the Methods section.*
☐	☒	A description of all covariates tested
☐	☒	A description of any assumptions or corrections, such as tests of normality and adjustment for multiple comparisons
☐	☒	A full description of the statistical parameters including central tendency (e.g. means) or other basic estimates (e.g. regression coefficient) AND variation (e.g. standard deviation) or associated estimates of uncertainty (e.g. confidence intervals)
☐	☒	For null hypothesis testing, the test statistic (e.g. *F*, *t*, *r*) with confidence intervals, effect sizes, degrees of freedom and *P* value noted<br>*Give P values as exact values whenever suitable.*
☐	☒	For Bayesian analysis, information on the choice of priors and Markov chain Monte Carlo settings
☒	☐	For hierarchical and complex designs, identification of the appropriate level for tests and full reporting of outcomes
☐	☒	Estimates of effect sizes (e.g. Cohen's *d*, Pearson's *r*), indicating how they were calculated

Our web collection on statistics for biologists contains articles on many of the points above.

Software and code

Policy information about availability of computer code

Data collection	Microsoft Excel and Access
Data analysis	NONMEM version 7.4 or R version 3.6.1, final model codes will be made available on github (10.5281/zenodo.5562807)

For manuscripts utilizing custom algorithms or software that are central to the research but not yet described in published literature, software must be made available to editors and reviewers. We strongly encourage code deposition in a community repository (e.g. GitHub). See the Nature Portfolio guidelines for submitting code & software for further information.

Data

Policy information about availability of data

All manuscripts must include a data availability statement. This statement should provide the following information, where applicable:

- Accession codes, unique identifiers, or web links for publicly available datasets
- A description of any restrictions on data availability
- For clinical datasets or third party data, please ensure that the statement adheres to our policy

All deidentified pharmacologic and epidemiologic data is available upon request to the corresponding author as not all individual level data has been approved to share by collaborators.

Field-specific reporting

Please select the one below that is the best fit for your research. If you are not sure, read the appropriate sections before making your selection.

- [x] Life sciences
- [ ] Behavioural & social sciences
- [ ] Ecological, evolutionary & environmental sciences

For a reference copy of the document with all sections, see nature.com/documents/nr-reporting-summary-flat.pdf

Life sciences study design

All studies must disclose on these points even when the disclosure is negative.

Sample size	All available participants from the parent clinical trial contributed data and were included (280 participants) in the analysis, and this determined the sample size.
Data exclusions	No data were excluded from the analysis.
Replication	Quality control measures were replicated with each set of piperaquine assays conducted. If the quality control samples did not provide <10% variability from prespecified piperaquine concentrations, then the concurrent assays were repeated using the same participant plasma samples. Replication of all plasma piperaquine concentrations in the study was not possible as very small blood volumes were collected to minimize harm for infant study participants and high costs of the assay. Similarly, for the genotyping of pfmdr1 and pfcrt conducted for this manuscript, repetition of a genotype was conducted if the control samples did not provide expected results or the assay did not provide a result. Replication of all results was not conducted to maintain costs within acceptable limits.
Randomization	Participants were randomized to DP every 12 weeks or DP every 4 weeks by a parent clinical trial and not for the purposes of this analysis. The parent clinical trial employed block randomization, with the exception that all children whose mothers received sulfadoxine-pyrimethamine received DP every 12 weeks. This was to maximize the power of the parent study to determine if maternal malaria chemoprevention regimen impacted childhood risk of malaria. To control for this, we evaluated maternal malaria chemoprevention regimen as a covariate in our PKPD model.
Blinding	All clinical and laboratory staff were blinded during collection of the clinical data and when obtaining the piperaquine concentrations and genotype data described in this study. The first and last authors were unblinded to assist in obtaining and reporting preliminary results but they were not involved in the clinical data collection.

Reporting for specific materials, systems and methods

We require information from authors about some types of materials, experimental systems and methods used in many studies. Here, indicate whether each material, system or method listed is relevant to your study. If you are not sure if a list item applies to your research, read the appropriate section before selecting a response.

Materials & experimental systems

n/a	Involved in the study	
x		Antibodies
x		Eukaryotic cell lines
x		Palaeontology and archaeology
x		Animals and other organisms
	x	Human research participants
	x	Clinical data
x		Dual use research of concern

Methods

n/a	Involved in the study	
x		ChIP-seq
x		Flow cytometry
x		MRI-based neuroimaging

Human research participants

Policy information about studies involving human research participants

Population characteristics	This study was conducted using blood samples collected from a previously conducted randomized controlled trial that was not conducted for the purposes of this study. The eligible population were neonates, born to mothers enrolled in a separate trial of IPT during pregnancy in Tororo, Uganda (NCT02163447), were enrolled at birth from October, 2014 to May, 2015, and followed for 36 months.
Recruitment	Neonates were recruited by the parent study investigators. Guardians of all children born to mothers from the parent IPT during pregnancy trial in Tororo, Uganda (NCT02163447) were invited to participate in the study in order to minimize enrollment bias.
Ethics oversight	The study protocol was approved by the Makerere University School of Biomedical Sciences Research and Ethics Committee, the Ugandan National Council for Science and Technology and the University of California, San Francisco Committee on Human Research.

Note that full information on the approval of the study protocol must also be provided in the manuscript.

Clinical data

Policy information about clinical studies

All manuscripts should comply with the ICMJEguidelines for publication of clinical research and a completedCONSORT checklist must be included with all submissions.

Clinical trial registration	Parent study - NCT02163447
Study protocol	https://clinicaltrials.gov/ct2/show/NCT02163447
Data collection	Data were collected from October, 2014 to May, 2015 from children enrolled in the study in Tororo, Uganda. The study clinic was located at the Infectious Diseases Research Collaboration Study Site at Tororo, District Hospital. All patient samples utilized for this analysis were collected at this study clinic. Plasma for piperaquine concentrations were shipped on dry ice to the UCSF Drug Research Unit in San Francisco, CA. Filter papers were transported at room temperature to UCSF, where parasite genotypes were obtained in the laboratory of Phil Rosenthal at UCSF Zuckerburg San Francisco General Hospital.
Outcomes	The primary outcome for this study was incident clinical malaria, defined by the parent study as fever and positive blood smear at least 14 days from a prior diagnosis of malaria. This has been the standard definition of malaria in malaria treatment and prevention studies. Secondary outcomes included the select genotypes at pfmdr1 or pfcrt which are associated with decreased aminoquinoline sensitivity, a mutant parasite at each locus (86, 184, or 1246) classified if a mutant parasite was detected in a sample, while wild type was defined as only wild type identified in the sample (please see the manuscript for full details of genotype definitions).
